# Voluntary Wheel Running Partially Compensates for the Effects of Global Estrogen Receptor-α Knockout on Cortical Bone in Young Male Mice

**DOI:** 10.3390/ijms22041734

**Published:** 2021-02-09

**Authors:** Rebecca K. Dirkes, Nathan C. Winn, Thomas J. Jurrissen, Dennis B. Lubahn, Victoria J. Vieira-Potter, Jaume Padilla, Pamela S. Hinton

**Affiliations:** 1 Nutrition and Exercise Physiology, University of Missouri, Columbia, MO 65211, USA; rdmr6@umsystem.edu (R.K.D.); nathan.winn@Vanderbilt.edu (N.C.W.); tjjmy9@umsystem.edu (T.J.J.); vieirapotterv@missouri.edu (V.J.V.-P.); padillaja@missouri.edu (J.P.); 2 Department of Molecular Physiology and Biophysics, Vanderbilt University, Nashville, TN 37203, USA; 3 Department of Biochemistry, University of Missouri, Columbia, MO 65211, USA; lubahnd@missouri.edu; 4 Child Health, University of Missouri, Columbia, MO 65211, USA; 5 Dalton Cardiovascular Research Center, University of Missouri, Columbia, MO 65211, USA

**Keywords:** estrogen, estrogen receptors, exercise, sclerostin, bone health

## Abstract

Estrogen receptor-α knockout (ERKO) in female, but not male, mice results in an impaired osteogenic response to exercise, but the mechanisms behind this ability in males are unknown. We explored the main and interactive effects of ERKO and exercise on cortical geometry, trabecular microarchitecture, biomechanical strength, and sclerostin expression in male mice. At 12 weeks of age, male C57BL/6J ERKO and WT animals were randomized into two groups: exercise treatment (EX) and sedentary (SED) controls, until 22 weeks of age. Cortical geometry and trabecular microarchitecture were measured via μCT; biomechanical strength was assessed via three-point bending; sclerostin expression was measured via immunohistochemistry. Two-way ANOVA was used to assess sclerostin expression and trabecular microarchitecture; two-way ANCOVA with body weight was used to assess cortical geometry and biomechanical strength. ERKO positively impacted trabecular microarchitecture, and exercise had little effect on these outcomes. ERKO significantly impaired cortical geometry, but exercise was able to partially reverse these negative alterations. EX increased cortical thickness regardless of genotype. There were no effects of genotype or exercise on sclerostin expression. In conclusion, male ERKO mice retain the ability to build bone in response to exercise, but altering sclerostin expression is not one of the mechanisms involved.

## 1. Introduction

Estrogen is one of the most influential hormones in growth and maintenance of the skeleton across the entire lifespan in both males and females [1,2,3,4,5]. Estrogen actions are mediated primarily by estrogen binding with the nuclear estrogen receptor (ER), which is found in two isoforms, ERα and ERβ. Bone expresses both ERα and ERβ; however, ERα tends to be more prevalent in cortical bone, whereas ERβ is more widely distributed in cancellous bone [6]. Global ERα knock out (ERKO) decreased longitudinal bone growth in both male and female mice [7,8,9]. Male ERKO mice show reduced bone turnover compared to wild-type (WT) controls [10], resulting in alterations in cortical bone geometry associated with decreased bending strength, such as decreased cortical bone area and cortical thickness [9,10]. However, cancellous bone mineral density (BMD) and total cancellous bone are increased in male ERKO mice, due to increased trabecular number [10,11]. These differential responses to the loss of ERα in cortical versus cancellous bone are most likely due to the variable distribution of estrogen receptors [6].

Estrogen and estrogen receptors in bone play an important role in the skeletal response to mechanical loading [12,13]. ERα is directly involved in the osteoblast response to strain [14,15,16], and ERα is upregulated and activated after application of mechanical strain. In vitro, osteoblasts taken from male and female ERKO mice do not proliferate and respond to strain like osteoblast cultures from WT controls [17], and female ERKO mice had an impaired osteogenic response to exercise [18]. However, male ERKO mice had either no differences or increased osteogenic response to mechanical loading compared to WT controls [19,20]. This implies some type of compensatory mechanism that allows for an osteogenic response to mechanical loading in ERKO males, possibly through ERβ or androgen receptors in bone [21].

Another recently hypothesized role of estrogen in the skeletal response to mechanical loading is the downregulation of the protein sclerostin. Osteocytes express sclerostin, which is a competitive inhibitor of the canonical Wnt signaling pathway. Canonical Wnt signaling plays a key role in bone formation, as it increases transcription of several osteogenic genes (reviewed in [22,23]). Briefly, association of the Wnt molecule with its receptor, co-receptor lipoprotein receptor-related protein 5 or 6 (LRP 5/6) leads to the release of a transcription factor, β-catenin, into the cytosol. β-catenin can then translocate into the nucleus, where it activates the transcription of several osteogenic genes, especially those related to osteoblast differentiation, such as osteoprotegerin. Inhibition of the Wnt signaling pathway via sclerostin is associated with decreased bone growth [24]. Animal and cell culture models show that sclerostin expression in osteocytes is regulated by several external factors, including insulin-like growth factor-I, parathyroid hormone, androgens and estrogens, and mechanical loading [24]. In healthy animals, mechanical loading downregulates sclerostin expression and leads to osteogenesis [24,25]. However, the specific role of estrogen in the control of sclerostin expression, and the effects of estrogen status on the sclerostin-mediated skeletal response to mechanical loading is still unknown.

In women and female rodent models, estrogen status is inversely related to sclerostin expression, whether measured via serum sclerostin levels [26,27,28] or by sclerostin mRNA [29] or protein in the bone [30,31]. In osteoblasts derived from female mice, sclerostin expression was downregulated after the application of exogenous estrogen, and ERβ appeared to play a greater role in the regulation of sclerostin than ERα [14]. In older men, estrogen treatment lowered circulating levels of sclerostin [32], implying that the inverse relationship between estrogen and sclerostin is also present in males. However, because circulating levels of sclerostin may not reflect bone sclerostin expression [33], further studies looking at the effect of estrogen on bone sclerostin expression in males are warranted.

The essential role of estrogen in bone health throughout the life cycle has been well-established in both sexes. However, questions still remain regarding the relative importance of specific estrogen receptors, especially ERα, in osteocyte sclerostin expression and the osteogenic response to exercise in males. Here, we look at the effects of global ERα knockout and voluntary wheel running exercise on cortical geometry, trabecular microarchitecture, biomechanical strength, and osteocyte sclerostin expression in male mice. We hypothesized that ERKO animals would have impaired cortical geometry and biomechanical strength compared to WT animals, but that exercise could lead to partial improvements in cortical geometry and biomechanical strength in ERKO animals, and significant improvements in the WT animals. We also hypothesized that ERKO animals would have increased trabecular bone volume compared to WT animals. Finally, we hypothesized that ERKO animals would have higher sclerostin expression than WT animals, and that exercise would downregulate sclerostin only in the WT groups.

## 2. Results

### 2.1. Animal Characteristics

The metabolic characteristics of these animals have been previously published as part of a larger study [34], and the results have been summarized in Table 1. Briefly, exercise significantly decreased body mass (*p* = 0.001) and body fat percentage (*p* = 0.004), and increased average weekly food intake (*p* = 0.001), regardless of genotype (Table 1). Exercise also significantly decreased LDL cholesterol, triglycerides, and leptin [34]. There was no difference in running distance between groups provided with running wheels [34]. ERKO animals had significantly higher LDL and total cholesterol compared to WT animals [34]. There was no effect of ERKO or exercise status on fasting blood glucose or insulin levels or HOMA-IR [34].

### 2.2. Tibial Cortical Geometry

**Figure 1:**
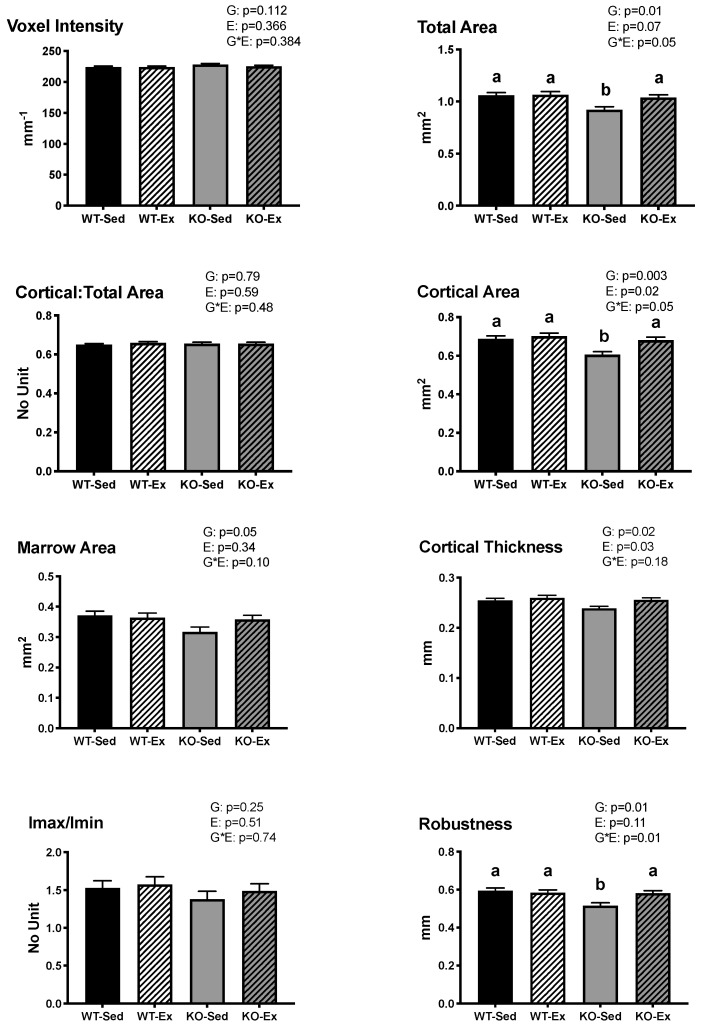
Tibial cortical geometry. Cortical geometry of the tibia. Data are adjusted means ± SEM, *n* = 6–8 per group ERKO: ERα knock out; WT: wild type; Ex: voluntary wheel running; Sed: sedentary. G: main effect of genotype (*p* ≤ 0.05); EX: main effect of exercise status (*p* ≤ 0.05); different letters (a,b) denote significance (*p* ≤ 0.05) if G*EX interaction is present. There was a significant main effect of G on Tt.Ar ((mm^2^): ERKO = 0.980 ± 0.019; WT = 1.063 ± 0.019; *p* = 0.007), Ct.Ar ((mm^2^): ERKO = 0.643 ± 0.011; WT = 0.695 ± 0.011; *p* = 0.003), Ma.Ar ((mm^2^): ERKO = 0.337 ± 0.010; WT = 0.368 ± 0.010; *p* = 0.05), Ct.Th ((mm): ERKO = 0.247 ± 0.003; WT = 0.257 ± 0.003; *p* = 0.02), and robustness ((no unit): ERKO = 0.55 ± 0.01; WT = 0.59 ± 0.01; *p* = 0.01). There was a significant main effect of EX on Ct.Ar ((mm^2^): Ex = 0.691 ± 0.011; Sed = 0.646 ± 0.011; *p* = 0.02) and Ct.Th ((mm): Ex = 0.258 ± 0.003; Sed = 0.247 ± 0.003; *p* = 0.03).

Tibial length was not different between groups. There was a main effect of genotype (*p* = 0.007) on Tt.Ar, with the ERKO animals having significantly lower Tt.Ar compared to the WT animals. There was a significant interaction (*p* = 0.05) between genotype and exercise, in that EX increased Tt.Ar only in the ERKO animals. ERKO animals had significantly lower robustness (Tt.Ar/Le) than WT counterparts (*p* = 0.01), but there was a significant interaction (*p* = 0.01) between genotype and exercise, in that EX increased robustness only in the ERKO animals. ERKO animals also had significantly lower Ma.Ar. than WT animals (*p* = 0.05). There was a main effect of genotype (*p* = 0.003) and exercise (*p* = 0.02) on Ct.Ar, with ERKO animals having lower Ct.Ar compared to WT and EX animals having higher Ct.Ar than SED animals. There was a significant interaction (*p* = 0.05) between genotype and exercise on Ct.Ar, in that EX increased Ct.Ar significantly more in the ERKO animals than in the WT animals. There were main effects of genotype (*p* = 0.02) and exercise (*p* = 0.03) on Ct.Th, with ERKO animals having lower Ct.Th than WT animals, and EX animals having higher Ct.Th than SED animals. There were no differences in Ct.Ar/Tt.Ar, Imax/Imin ratio, or voxel intensity between groups (Figure 1). When body weight was not used as a covariate, the significant effect of genotype on Tt.Ar, Ma.Ar, Ct.Ar remained, but the differences in Ct.Th were no longer significant, and there were no longer significant effects of exercise (data not shown).

### 2.3. Tibial Biomechanical Strength

**Figure 2:**
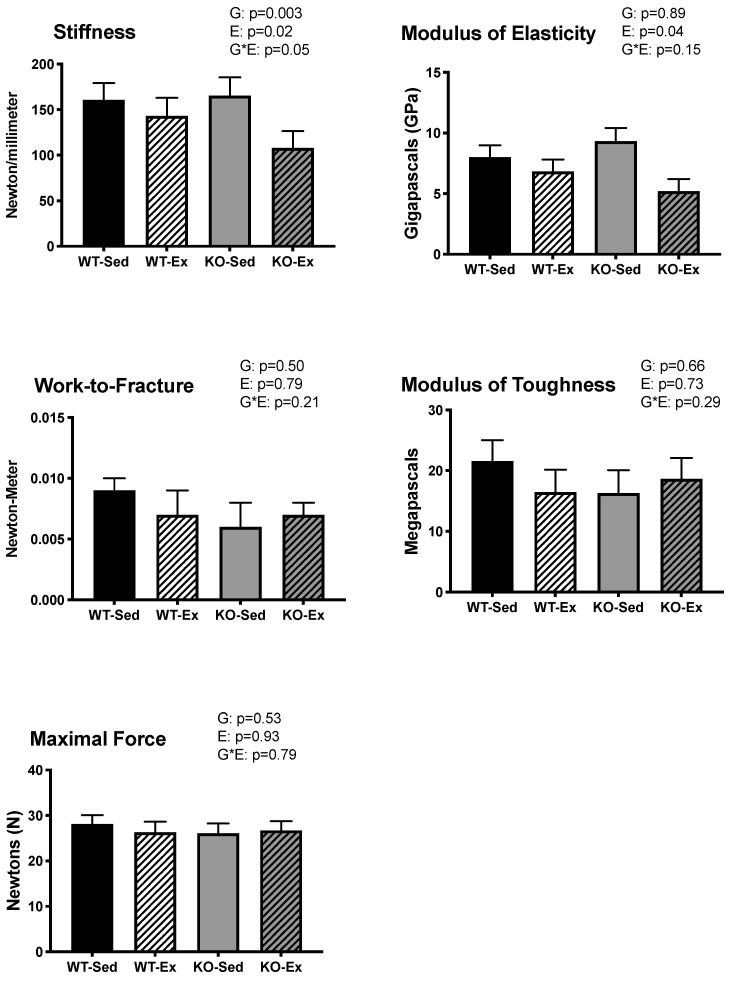
Biomechanical properties. Biomechanical strength of the tibia. Data are adjusted means ± SEM, *n* = 6–8 per group ERKO: ERα knock out; WT: wild type; Ex: voluntary wheel running; Sed: sedentary. G: main effect of genotype (*p* ≤ 0.05); EX: main effect of exercise status (*p* ≤ 0.05). There was a significant main effect of EX on Young’s modulus of elasticity ((GPa): Ex = 6.012 ± 0.762; Sed = 8.665 ± 0.762; *p* = 0.04).

Exercising animals had significantly lower Young’s modulus of elasticity than their sedentary counterparts (*p* = 0.04), regardless of genotype. There were no differences between groups in max force, stiffness, work-to-fracture, or modulus of toughness. (Figure 2) When body weight was not used as a covariate, there were no differences between groups in any biomechanical outcomes (data not shown).

### 2.4. Tibial Trabecular Microarchitecture

**Figure 3:**
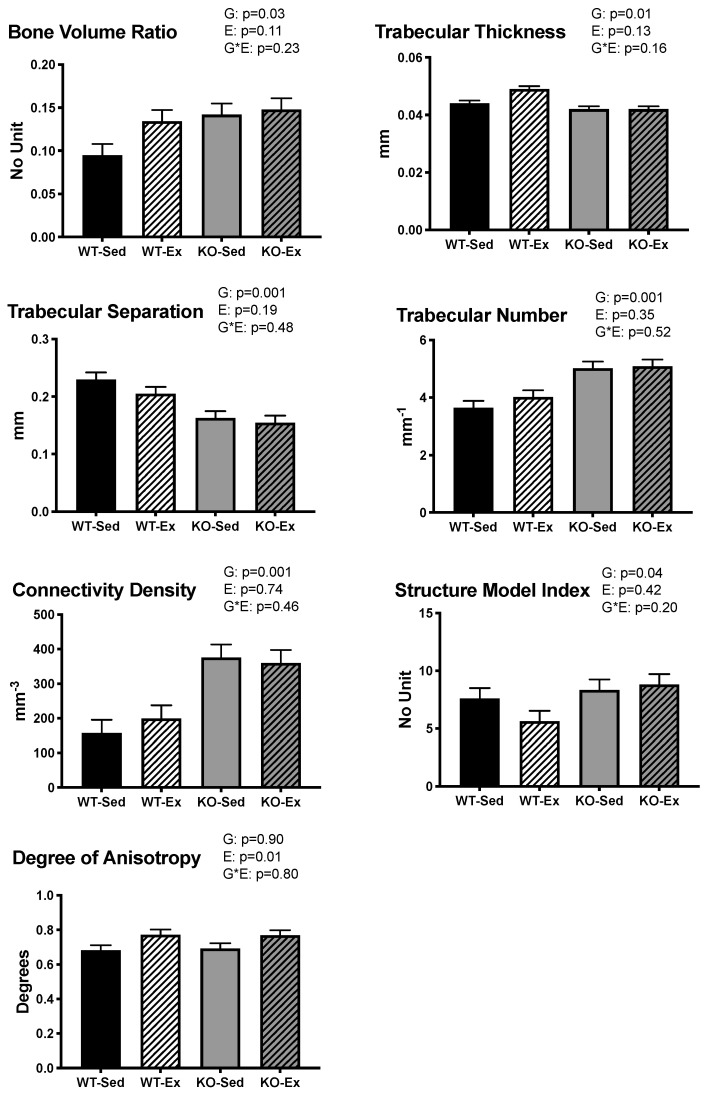
Tibial trabecular microarchitecture. Trabecular microarchitecture of the tibia. Data are means ± SEM, *n* = 6–8 per group ERKO: ERα knock out; WT: wild type; Ex: voluntary wheel running; Sed: sedentary. G: main effect of genotype (*p* ≤ 0.05); EX: main effect of exercise status (*p* ≤ 0.05). There was a significant main effect of G on BV/TV ((no unit): ERKO = 0.145 ± 0.01; WT = 0.114 ± 0.01; *p* = 0.03), Tb.Th ((mm): ERKO = 0.042 ± 0.001; WT = 0.047 ± 0.001; *p* = 0.006), Tb.Sp ((mm): ERKO = 0.159 ± 0.01; WT = 0.218 ± 0.01; *p* = 0.001), Tb.N ((mm^−1^): ERKO = 5.06 ± 0.163; WT = 3.84 ± 0.16; *p* = 0.001), Conn.D ((mm^3^): ERKO = 367.7 ± 26.9; WT = 178.5 ± 26.9; *p* = 0.001), and SMI ((no unit): ERKO = 8.58 ± 0.64; WT = 6.62 ± 0.64; *p* = 0.04). There was a significant main effect of EX on DA ((degree): Ex = 0.771 ± 0.021; Sed = 0.688 ± 0.021; *p* = 0.01).

There was a main effect of genotype on BV/TV (*p* = 0.034), Tb.N (*p* = 0.001), Conn.D (*p* = 0.001), and SMI (*p* = 0.04), with ERKO animals having higher values than WT animals in these outcomes. There was a main effect of genotype on Tb.Th (*p* = 0.006) and Tb.Sp (*p* = 0.001), with ERKO animals having lower values than WT animals. Exercising animals had significantly higher DA than their sedentary counterparts (*p* = 0.01), regardless of genotype. (Figure 3)

### 2.5. Femoral Osteocyte Sclerostin Expression

**Figure 4:**
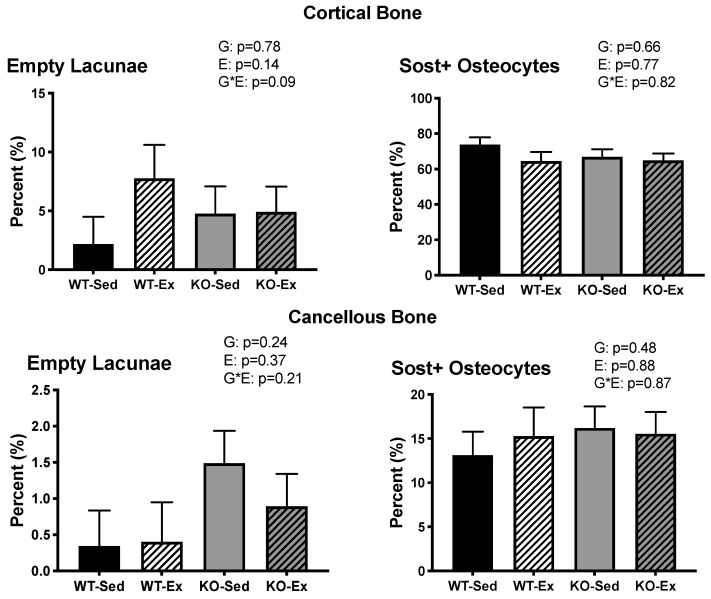
Sclerostin expression. Sclerostin expression of the femur in cortical and cancellous bone. Data are means ± SEM, *n* = 6–8 per group ERKO: ERα knock out; WT: wild type; Ex: voluntary wheel running; Sed: sedentary.

There were no differences in percent empty lacunae or percent sclerostin-positive osteocytes between groups in samples of either cortical or cancellous bone. (Figure 4)

## 3. Discussion

Here we showed that ERKO had differential effects on cortical and cancellous bone in young male mice compared to their WT counterparts. More specifically, ERKO animals, regardless of exercise status, had improved measures of trabecular microarchitecture, such as bone volume and trabecular number; however sedentary ERKO animals had significant negative alterations in cortical bone geometry, such as reduced cortical thickness and area relative to controls. We also found that exercise begun after skeletal maturity could reverse some of these negative alterations in cortical bone of ERKO mice. Exercising male ERKO animals had significantly improved measures of cortical geometry than their sedentary counterparts, implying that ERα is not necessary for males to grow bone in response to exercise. Exercise, regardless of genotype, decreased Young’s modulus of elasticity in the tibia, but there was no effect of exercise or genotype on any other measures of biomechanical strength or sclerostin expression in cortical or cancellous bone of the femur.

The negative morphological changes seen in the cortical bone of male, global ERKO animals in this study are supported by previous studies both by ourselves [35] and others [9,10,11]. Estrogen and estrogen receptors have significant, cell-specific roles in the development and maintenance of bone mass—estrogen blocks osteocyte and osteoblast apoptosis, induces osteoblast differentiation, increases osteoclast apoptosis, and reduces osteoclast differentiation [36,37,38,39,40]. ERα is particularly important in the maintenance of cortical bone mass due to its higher prevalence compared to ERβ [6] and its actions in certain cell types. When ERα was deleted from osteoblast precursors, femoral BMD and cortical thickness were reduced compared to wild-type animals [41], whereas there were no alterations in cortical bone when ERα was deleted from mature osteocytes only [42]. This indicates that ERα controls cortical bone formation through actions on progenitor cells and supports our findings in this global knockout model.

Previous studies have shown that both global [19] and osteoblast-specific [20] male ERKO models can respond to exercise despite the loss of ERα. This is in direct contrast to females, which require ERα for an osteogenic response to exercise [18]. To the authors’ knowledge, we were the first to further explore one cellular mechanism of the exercise response by analyzing sclerostin expression in male ERKO animals. We have previously shown that aged, sedentary male ERKO animals have higher sclerostin expression than their WT counterparts [35]. Considering that exercise downregulates sclerostin [24], we wanted to explore whether exercise-induced decreases in sclerostin are estrogen-mediated. While exercise decreased sclerostin expression in both groups, it was not statistically significant. There were no differences between ERKO and WT in sclerostin expression, which implies that there is no effect of ERKO on sclerostin expression in younger males. The differences between this study and our previous study [35] could be partially explained by age or metabolic status. In our previous study, ERKO animals had higher fasting blood glucose levels than their WT counterparts, and high blood glucose is associated with increased sclerostin expression [24]. In this study, there were no difference in blood glucose, which could partially explain the lack of differences in sclerostin expression. In addition, sclerostin naturally increases with age [24], and this higher expression overall could exacerbate differences triggered by metabolic changes over time. However, more studies are needed to fully understand the interaction of ERα, age, and exercise on sclerostin expression.

Exercise had minimal effects in the cortical bone of WT animals. Exercise increased cortical thickness but did not have a significant effect on measures of area or robustness. We attribute this to the initiation of exercise after skeletal maturity—exercise began at 12 weeks of age, which is around skeletal maturity [43]. Previous studies have shown that bone cells are less responsive to exercise after skeletal maturity, and it thus takes higher mechanical forces to induce osteogenesis [44]. However, in the ERKO animals, which had significant impairments in cortical bone geometry, exercise induced enough strain for a significant osteogenic response. This could partially be due to the increased body weight in exercising ERKO animals compared to exercising WT animals, since higher body weight would result in higher strains [45]. This also could be an indicator that ERα limits the sensitivity of bone to exercise, and the removal of ERα allows for a more robust osteogenic response. Exercising ERKO animals had significantly higher cortical and total area, cortical thickness, and robustness than their sedentary counterparts, which equalized their cortical geometry to that of the WT animals. Interestingly, exercising animals had lower Young’s modulus of elasticity and stiffness, although stiffness was not quite at a significant level (*p* = 0.10), regardless of genotype. Both are measures of the elasticity of the bone, or the ability of the bone to resist deformation before fracture, but stiffness is a measure of the elasticity of the bone structure whereas Young’s modulus is a measure of the elasticity of the bone material. There was no effect of exercise or genotype on maximal load sustained, suggesting that these alterations in stiffness and elasticity did not have a negative impact on overall strength of the bone. The differences in Young’s modulus without corresponding differences in cortical geometry could indicate differences in bone quality. While we saw no differences in voxel intensity, a proxy measure for tissue mineral density, this difference in Young’s modulus would warrant further exploration of tissue-level measures of bone quality in the future. It should also be acknowledged that tibiae have an irregular cross-sectional shape, which makes them less suitable for a three-point bending test than a tubular bone, such as the radius or femur [46], and this higher variability in results could contribute to the lack of statistical differences.

The positive morphological changes in the trabecular bone of the ERKO animals have also been supported in previous studies by both ourselves [35] and others [10,11]. These improvements can be explained by the greater presence of ERβ in cancellous bone. In cancellous bone, ERβ and ERα appear to have similar actions, and both play a role in maintaining total bone mass [40]. Thus, the loss of ERα does not lead to a loss of bone mass as it does in cortical bone. In fact, our results could indicate that ERα plays a more regulatory or restrictive role in trabecular bone, considering that the loss of ERα increases measures of trabecular bone mass. However, we were one of the first to explore the effects of exercise on trabecular microarchitecture in ERKO mice, as well as further explore the cellular mechanisms by measuring sclerostin expression. Exercise begun after skeletal maturity increased the degree of anisotropy, which is a strong indicator of trabecular bone strength [47]. Thus, it is possible that exercise increased the strength of the trabecular bone in conjunction with the morphological changes; however, we did not have the capacity to measure trabecular bone strength. We saw no effect of ERKO or exercise on sclerostin expression in cancellous bone, consistent with previous studies [35].

In conclusion, we found that ERKO has differential effects on cortical and cancellous bone in young, male mice. Specifically, ERKO significantly improved trabecular microarchitecture, implying ERα is not required for the maintenance of trabecular bone. However, ERKO negatively impacted cortical geometry, which supports a significant role for ERα in the maintenance of cortical bone in sedentary animals. We also found that exercise started after skeletal maturity was able to reverse the negative alterations in cortical geometry in the ERKO animals. This would indicate that ERα is not necessary for an osteogenic response to mechanical loading in male mice. There were no effects of ERKO or exercise on sclerostin expression, and thus further studies are warranted to explore additional cellular mechanisms which allow this osteogenic response to exercise in male ERKO animals and if these mechanisms can translate to humans. This would allow for further understandings of potential targets to improve the efficacy of exercise treatments for osteoporosis and other diseases of low bone mass in both men and women.

## 4. Materials and Methods

### 4.1. Experimental Design

This study is part of a larger study investigating the effects of global ERKO on glycemic control, inflammation, and hepatic steatosis in male mice, and whether exercise could be an effective treatment to reverse the negative impacts of ERKO [34]. Heterozygote ERα−/+ mice on a C57BL/6J background were bred at our facility to produce male homozygote (ERα−/−) and littermate wild-type mice, as previously described [48,49]. Briefly, development of the ERα−/− mouse was accomplished by homologous recombination and insertion of a neomycin sequence containing premature stop codons and polyadenylation sequences into a Not1site in exon 2 of the mouse estrogen receptor gene [48,49,50]. After weaning, mice were fed standard rodent chow ((3.3 kcal/g of food), 13% kcal fat, 57% kcal carbohydrate, and 30% kcal protein, 5001, LabDiet, St. Louis, MO, USA) ad libitum until 12 weeks of age. At 12 weeks of age, all mice were given ad libitum access to a high-fat diet (4.65 kcal/g of food; 46.0% kcals from fat, 36.0% kcals from carbohydrate with sucrose content (per weight) of 17.5% and high-fructose corn syrup content of 17.5% (Test Diet modified 58Y1; 5APC)), and both ERα−/− (ERKO) and wild-type (WT) animals were randomized into two groups—an exercising (EX) group given access to running wheels, and a sedentary (SED) control group with no running wheels—resulting in four experimental groups: ERKO-EX, ERKO-SED, WT-EX, and WT-SED (*n* = 6–8/group). All mice were pair-housed (mixed genotypes) in a temperature-controlled environment at 25 °C, with a 0700–1900 light, 1900–0700 dark cycle. Body weight and food intake were measured weekly, and body composition was measured by a nuclear magnetic resonance imaging whole-body composition analyzer (EchoMRI 4in1/1100; Echo Medical Systems, Houston, TX, USA) on conscious mice one week prior to sacrifice. At 22 weeks of age, mice were euthanized following a 5 h fast. Blood samples were collected via cardiac puncture and centrifuged; plasma was separated, frozen in liquid nitrogen, and stored at −80 °C for further analysis. Circulating estradiol and fasting insulin, glucose, and lipids were measured as previously described [34]. Hindlimbs were harvested, wrapped in PBS-soaked gauze, frozen in liquid nitrogen, and stored at −80 °C for further analysis. All procedures were approved in advance by the University of Missouri Institutional Animal Care and Use Committee.

### 4.2. Tibial Cortical Geometry and Trabecular Microarchitecture

Micro-computed tomographic (µCT) imaging of the right tibia was performed using a high-resolution imaging system (Xradia 520 Versa, ZEISS, Oberkochen, Germany). The methods used were in accordance with guidelines for the use of µCT in rodents [51]. Scans were acquired using an isotropic voxel size of 0.012 mm, a peak X-ray tube potential of 60 kV, and 2 s exposure time. Trabecular bone microarchitecture was evaluated in a 0.5 mm region of interest directly below the growth plate of the proximal tibia, as previously described [52,53]. Cortical bone cross-sectional geometry was evaluated at a 1 mm region of interest at the mid-diaphysis of the tibia, or the midway point between the tibial crest and the tibiofibular joint, as previously described [52,53]. The optimized threshold function was used to delineate mineralized bone from soft tissue. Scans were analyzed using BoneJ software [54] (NIH public domain), and measures of cortical geometry and trabecular microarchitecture were collected. Outcomes for cortical geometry included tibia length (Le), total cross-sectional area inside the periosteal envelope (Tt.Ar), marrow area (Ma.Ar), cortical bone area (Ct.Ar), cortical area fraction (Ct.Ar/Tt.Ar), mean cortical thickness (Ct.Th), and robustness (R, total bone area over length calculated as R = Tt.Ar/Le). Voxel gray-scale intensity of the cortical bone was measured as a proxy for tissue mineral density. Outcomes for trabecular microarchitecture included bone volume fraction (BV/TV), connectivity density (Conn.D, degree of trabeculae connectivity normalized to total bone volume), mean trabecular thickness (Tb.Th), trabecular separation (Tb.Sp, distance between trabeculae), trabecular number (Tb.N, average number of trabeculae per unit length calculated as 1/(Tb.Th + Tb.Sp) [55]), structural model index (SMI), and degree of anisotropy (DA).

### 4.3. Tibial Biomechanical Strength

Biomechanical strength of the right tibia was performed using three-point bending [56]. Briefly, tibias were cleaned of all soft tissue and placed in the three-point bending apparatus with a span of 6 mm. Tibiae were loaded via a materials testing machine (Instron 5942; Instron, Inc., Norwood, MA, USA) at a rate of 10 mm/minute at the midpoint of the tibia until fracture. Outputs from the Instron machine were used to produce a load-displacement curve. The slope of the load-displacement curve was used to estimate material stiffness, and the area under the load-displacement curve was used to estimate work-to-fracture [57]. Maximal load was measured as the highest force applied to the bone before fracture [57]. Load-displacement data were converted into stress and strain to produce a stress-strain curve using the geometric measurements of the bone and following the equations of Turner and Burr [57]. The slope of the stress-strain curve was used to estimate Young’s modulus of elasticity, and the area under the curve was used to estimate the modulus of toughness [57].

### 4.4. Femoral Osteocyte Sclerostin Expression

The right femurs were fixed in 10% formalin for 48 h at 4 °C and then decalcified in 14% EDTA at 4 °C. Decalcified femurs were embedded in paraffin wax blocks, and 5 μm sections were taken transversely at the mid-diaphysis and the proximal metaphysis above the growth place of the femur, for measures of cortical and cancellous bone, respectively. The sections were deparaffinized and underwent heat-induced epitope retrieval overnight at 60 °C using a 10 mM sodium citrate buffer, followed by blocking of endogenous avidin and biotin expression (Avidin Biotin Blocking Solution, Thermo Scientific, Waltham, MA, USA). Sections were then incubated in anti-sclerostin primary antibodies (Abcam, Cambridge, UK) overnight at 4 °C, followed by blocking of endogenous peroxidase activity (3% H_2_O_2_, Ricca Chemical, Arlington, TX, USA) and secondary antibody application. Secondary antibody binding and detection were accomplished using a Vectastain Elite ABC kit (Vector Laboratories, Burlingame, CA, USA), with diaminobenzidine (ImmPACT DAB, Vector Laboratories, Burlingame, CA, USA) as the chromogen. Sections were counterstained with hematoxylin (Fisher Scientific, Hampton, NH, USA), dried, and mounted. Sections were analyzed at 20× for sclerostin expression. Sclerostin positive (Sost+) osteocytes were defined as osteocytes exhibiting brown staining, and sclerostin negative (Sost-) osteocytes were defined as osteocytes exhibiting blue (hematoxylin) staining. Data are reported as percent Sost+ osteocytes. In addition to Sost+ and Sost− osteocytes, empty osteocytic lacunae revealed by hematoxylin staining were counted, and data are reported as percent empty lacunae, as previously described [58].

### 4.5. Statistical Analysis

Two-way ANOVA was used to assess the main and interactive effects of genotype and exercise on metabolic outcomes, trabecular microarchitecture, and percentage of sclerostin+ osteocytes. Body weight is a strong predictor of cortical bone size and strength, so cortical geometry and biomechanical strength outcomes were assessed by two-way ANCOVA with final body weight included as a covariate [56]. We also ran two-way ANOVAs on cortical outcomes to confirm that differences body weight were a main driver in cortical differences. If an interaction was present, one-way ANOVA or ANCOVA was used as necessary to determine the location of the interaction. Data are presented as means ± SEM or adjusted means ± SEM. Statistical significance was set at *p* < 0.05. All analyses were performed using SPSS software (SPSS/25.0, SPSS, Chicago, IL, USA).

## Figures and Tables

**Table 1:** Metabolic characteristics.

	WT		ERKO		
Outcome	SED	EX	SED	EX	*p*-Value
Body Mass (g)	39.63 ± 1.70	33.06 ± 1.77 *	41.52 ± 1.70	36.39 ± 1.58 *	G: 0.129 E: 0.001 G*E: 0.670
Body Fat (%)	32.28 ± 2.33	22.96 ± 2.64 *	33.22 ± 2.33	27.56 ± 2.21 *	G: 0.254 E: 0.004 G*E: 0.450
Average Food Intake (g/week)	9.09 ± 0.21	10.20 ± 0.24 *	8.83 ± 0.21	9.60 ± 0.20 *	G: 0.054 E: 0.001 G*E: 0.432

Data are means ± SEM, *n* = 6–8 per group. ERKO: ERα knock out; WT: wild type; EX: voluntary wheel running; SED: sedentary. G: genotype; E: exercise status; G*E: genotype by exercise interaction. * *p* ≤ 0.05 vs. SED of same genotype. There was a significant main effect of EX on body mass ((g): EX = 34.73 ± 1.19; SED = 40.58 ± 1.18; *p* = 0.001), body fat ((%): EX = 25.26 ± 1.72; SED = 32.75 ± 1.65; *p* = 0.004), and average food intake ((g/wk): EX = 9.89 ± 0.15; SED = 8.96 ± 0.15; *p* = 0.001).

## Data Availability

Data available upon request.
